# Imeglimin exerts favorable effects on pancreatic β-cells by improving morphology in mitochondria and increasing the number of insulin granules

**DOI:** 10.1038/s41598-022-17657-3

**Published:** 2022-08-02

**Authors:** Junpei Sanada, Atsushi Obata, Yoshiro Fushimi, Tomohiko Kimura, Masashi Shimoda, Tomoko Ikeda, Yuka Nogami, Yoshiyuki Obata, Yuki Yamasaki, Shuhei Nakanishi, Tomoatsu Mune, Kohei Kaku, Hideaki Kaneto

**Affiliations:** Department of Diabetes, Endocrinology and Metabolism, Kawasaki Medical School, 577 Matsushima, Kurashiki, 701-0192 Japan

**Keywords:** Endocrinology, Medical research

## Abstract

Imeglimin is a new anti-diabetic drug commercialized in Japan (Twymeeg®) and has been drawing much attention in diabetes research area as well as in clinical practice. In this study, we evaluated the effect of imeglimin on pancreatic β-cells. First, single-dose administration of imeglimin enhanced insulin secretion from β-cells and decreased blood glucose levels in type 2 diabetic db/db mice. In addition, single-dose administration of imeglimin significantly augmented insulin secretion in response to glucose from islets isolated from non-diabetic db/m mice. Second, during an oral glucose tolerance test 4-week chronic treatment with imeglimin enhanced insulin secretion and ameliorated glycemic control in diabetic db/db mice. Furthermore, the examination with electron microscope image showed that imeglimin exerted favorable effects on morphology in β-cell mitochondria and substantially increased the number of insulin granules in type 2 diabetic db/db and KK-Ay mice. Finally, imeglimin reduced the percentage of apoptotic β-cell death which was accompanied by reduced expression levels of various genes related to apoptosis and inflammation in β-cells. Taken together, imeglimin directly enhances insulin secretion in response to glucose from β-cells, increases the number of insulin granules, exerts favorable effects on morphology in β-cell mitochondria, and reduces apoptotic β-cell death in type 2 diabetic mice, which finally leads to amelioration of glycemic control.

## Introduction

The number of subjects with type 2 diabetes mellitus is increasing worldwide due to lifestyle changes such as increase of fat intake and decrease of exercise. It is thought that the number of diabetes patients reflects an increase of overweight and obesity^1^. It is well known that inadequate glycemic control finally causes micro and macrovascular complications, which further leads to the decline in actives of daily life and increase of mortality.

Hyperglycemia is caused by increase of insulin resistance in insulin target tissues and/or decrease of insulin secretion from pancreatic β-cells. Increase of insulin resistance is associated with accumulation of visceral fat and/or decrease of physical activity. Decrease of insulin secretion in type 2 diabetes mellitus is often accompanied by decrease of pancreatic β-cell mass^2^. Indeed, it was reported that β-cell mass was reduced by 30% in subjects with type 2 diabetes mellitus compared with healthy subjects^3^.

It is known that apoptosis is involved in the reduction of β-cell mass^2–4^. This phenomenon is induced by alteration of various factors such as inflammation^5,6^, oxidative stress^7^ and endoplasmic reticulum (ER) stress^8^ due to chronic hyperglycemia. In addition, it is known that β-cells have less expression of antioxidant enzymes such as catalase and superoxide dismutase (SOD) compared to other organs. Thereby, β-cells are particularly vulnerable to oxidative stress induced by hyperglycemia. Therefore, it is very important to obtain adequate glycemic control to protect β-cells from oxidative stress. It has been reported so far that β-cell apoptosis is reduced by the treatment with anti-diabetic drugs in human and rodents^9–12^.

Imeglimin is a new anti-diabetic drug commercialized in Japan (Twymeeg®), and clinical trials with imeglimin in human subjects (TIMES 1–3) have shown its efficacy and safety of long-term therapy and its effectiveness of combination with other oral anti-diabetic drugs or insulin therapy^13–15^. The difference from the older anti-diabetic drugs is that the effects of imeglimin can be expected in various aspects. Several effects of imeglimin have been reported in animal experiments. First, imeglimin increases insulin secretion in response to glucose^16,17^, preserves β-cell mass^18^, and reduces pancreatic β-cell apoptosis by modulating the ER stress^19^. Second, imeglimin improves insulin sensitivity and inhibits glycogenesis^20,21^. In addition, it was reported that imeglimin prevented heart failure with preserved ejection fraction^22^ and ameliorated cardiac dysfunction in Zucker fa/fa rats^23^. Finally, imeglimin alleviates mitochondrial dysfunction in pancreatic β-cells and the liver^20,21^. However, the reports of these effects are still limited, and the mechanisms are not yet fully unknown.

In this study, we evaluated the effect of imeglimin on pancreatic β-cells using diabetic model mice such as db/db and KK-Ay mice and pancreatic islets isolated from diabetic and non-diabetic mice.

## Results

### Effects of single-dose administration of imeglimin on insulin secretion from pancreatic β-cells in type 2 diabetic and non-diabetic mice

**Figure 1:**
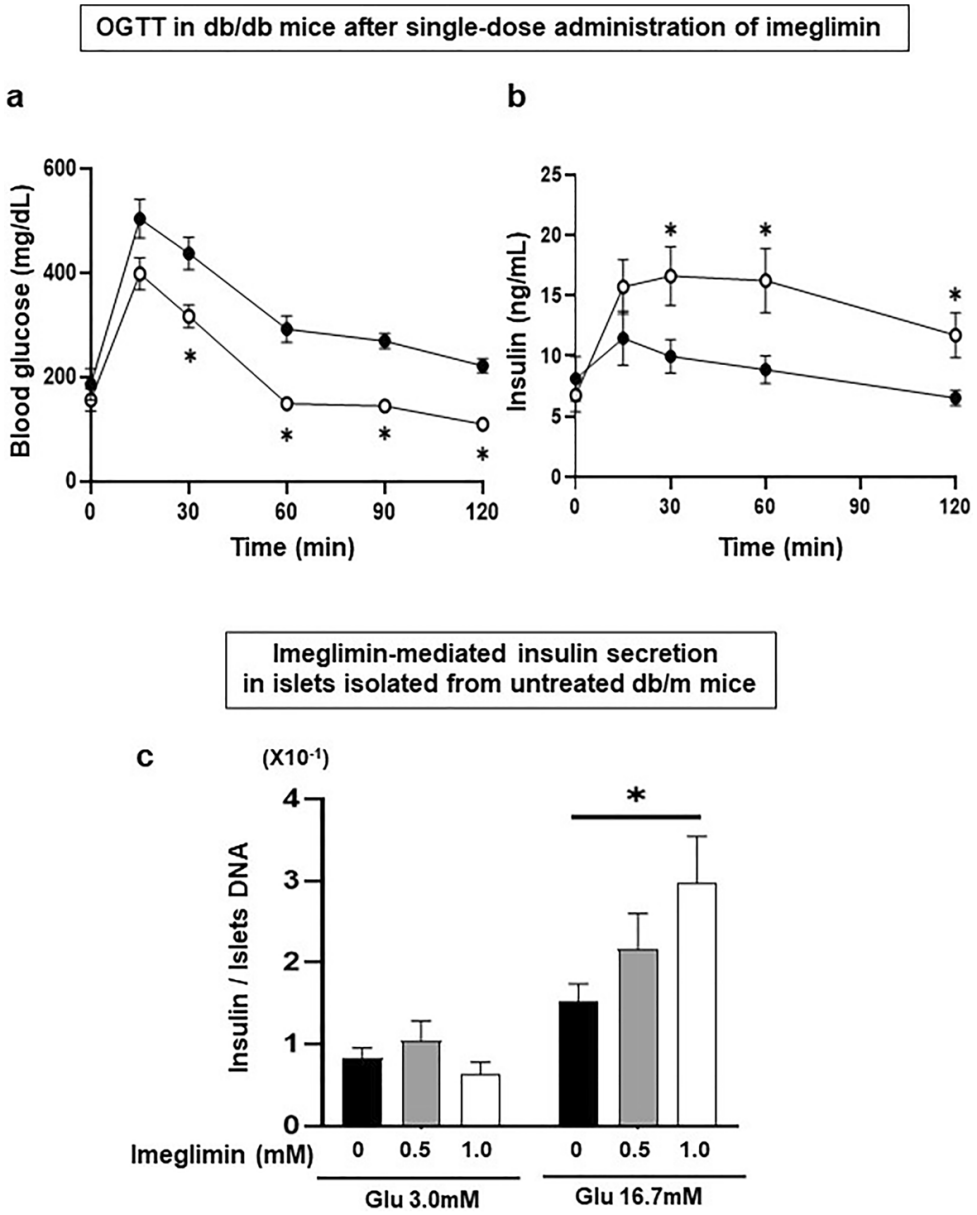
(**a**, **b**) Effects of single-dose administration of imeglimin on blood glucose and serum insulin levels in obese type 2 diabetic db/db mice. In oral glucose tolerance test after single-dose administration of imeglimin in 7-week-old db/db mice, blood glucose levels were significantly lower in the mice treated with imeglimin together with significant increase of insulin secretion compared to those without the treatment (control: black circle, imeglimin: white circle, n = 6). (**c**) Effects of single-dose administration of imeglimin on glucose-stimulated insulin secretion in pancreatic islets isolated from non-diabetic db/m mice. Insulin secretion was significantly augmented by imeglimin in a dose-dependent manner under a high glucose concentration, but not in a low glucose concentration, in islets isolated from db/m mice (db/m mice, n = 9–10). Values are the mean ± SEM of data obtained from each group. *p < 0.05.

To examine the effects of single-dose administration of imeglimin, we performed oral glucose tolerance test after single-dose administration of imeglimin in 7-week-old obese type 2 diabetic db/db mice. As shown in Fig. 1a, blood glucose levels were significantly lower in the mice treated with imeglimin. Also, as shown in Fig. 1b, serum insulin levels were significantly higher at 30 min and this effect was preserved until 120 min in the mice treated with imeglimin compared to those without treatment.

In addition, to further examine the effects of single-dose administration of imeglimin on insulin secretion, we evaluated glucose-stimulated insulin secretion after single-dose administration of imeglimin using pancreatic islets isolated from non-diabetic db/m mice. As shown in Fig. 1c, imeglimin significantly augmented insulin secretion in a dose-dependent manner under a high glucose concentration, but not in a low glucose concentration.

### Favorable effects of chronic treatment with imeglimin on pancreatic β-cell function and glycemic control in type 2 diabetic mice

**Figure 2:**
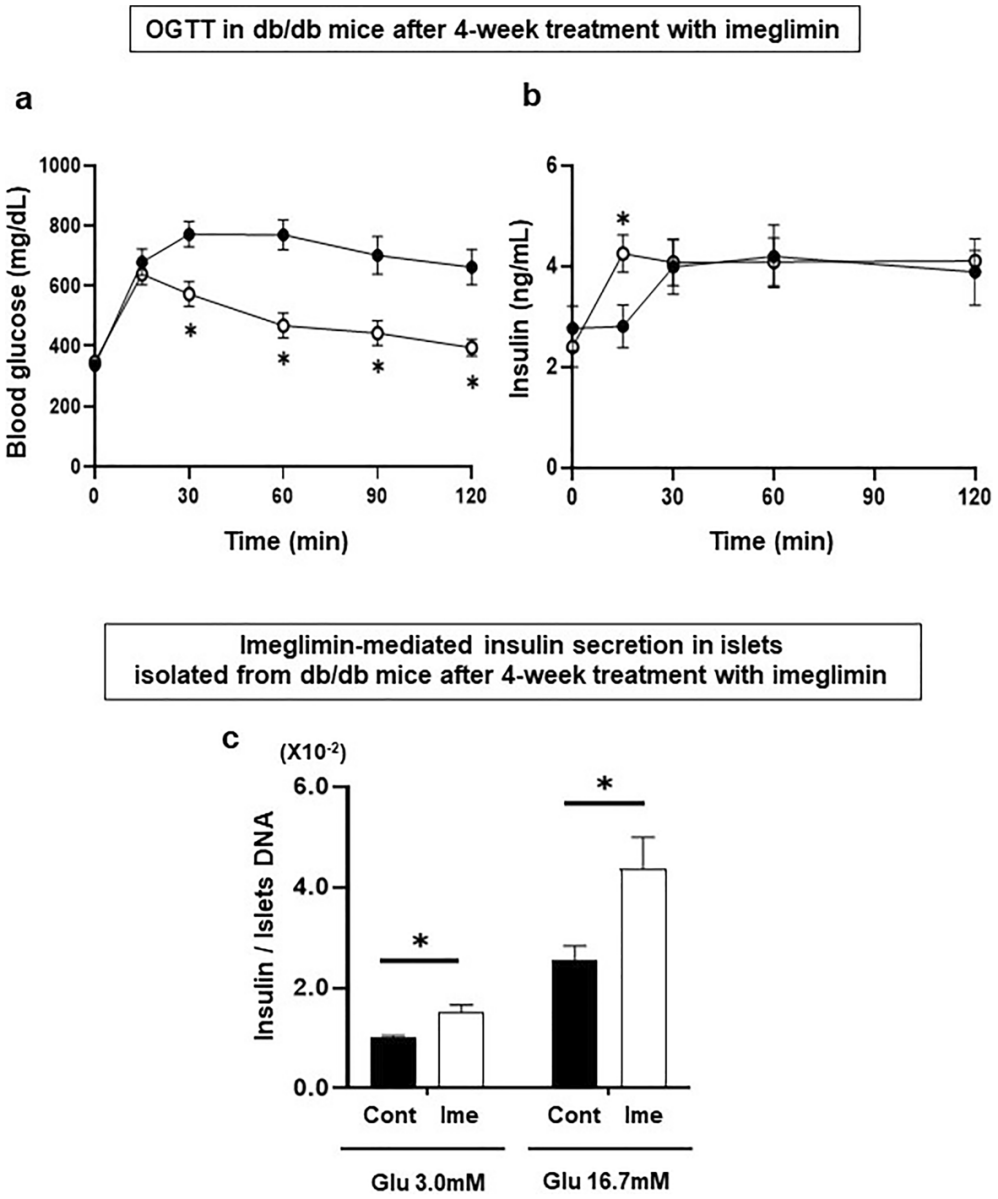
(**a**, **b**) Effects of chronic administration of imeglimin on blood glucose and serum insulin levels in obese type 2 diabetic db/db mice. In oral glucose tolerance test after 4-week administration of imeglimin in db/db mice, blood glucose levels were significantly lower in the mice treated with imeglimin together with significant increase of insulin secretion compared to those without the treatment (control: black circle, imeglimin: white circle, n = 6). (**c**) Glucose-stimulated insulin secretion in islets isolated from db/db mice after 4-week treatment with imeglimin. Insulin secretion was significantly increased in the mice treated with imeglimin compared to those without the treatment (n = 8). Values are the mean ± SEM of data obtained from each group. *p < 0.05.

To examine the chronic effects of imeglimin on pancreatic β-cell function and glycemic control, we treated obese type 2 diabetic db/db mice with imeglimin for 4 weeks (from 7 weeks old to 11 weeks old). As shown in Fig. 2a, blood glucose levels were significantly lower in the mice treated with imeglimin, compared to those without treatment, and their glucose levels were decreased to the same level before the loading. Also, as shown in Fig. 2b, serum insulin level was significantly higher at 15 min in the mice treated with imeglimin compared to that without treatment.

To further assess the effect of imeglimin on β-cell function, we evaluated glucose-stimulated insulin secretion using islets isolated from db/db mice after 4-week treatment with imeglimin. As shown in Fig. 2c, insulin secretion was significantly increased in islets from mice treated with imeglimin compared to those without treatment.

Next, to evaluate the possible influence of imeglimin on insulin sensitivity, we performed insulin tolerance test after the treatment with imeglimin in diabetic db/db and KK-Ay mice. As shown in Supplemental Fig. 1, there was no difference in blood glucose levels between with and without imeglimin treatment, suggesting that imeglimin does not show overt effect on insulin sensitivity although we should be aware that insulin tolerance test does not necessarily detect all influence on insulin sensitivity.

In addition, we evaluated body weight, serum total cholesterol and triglyceride levels before and after the treatment with imeglimin in non-diabetic db/m mice and type 2 diabetic db/db and KK-Ay mice. As shown in Supplemental Fig. 2, there were no differences in body weight, total cholesterol and triglyceride levels between with and without imeglimin treatment.

### Favorable effects of chronic treatment with imglimin on morphology in β-cell mitochondria and the number of insulin granules in type 2 diabetic mice

**Figure 3:**
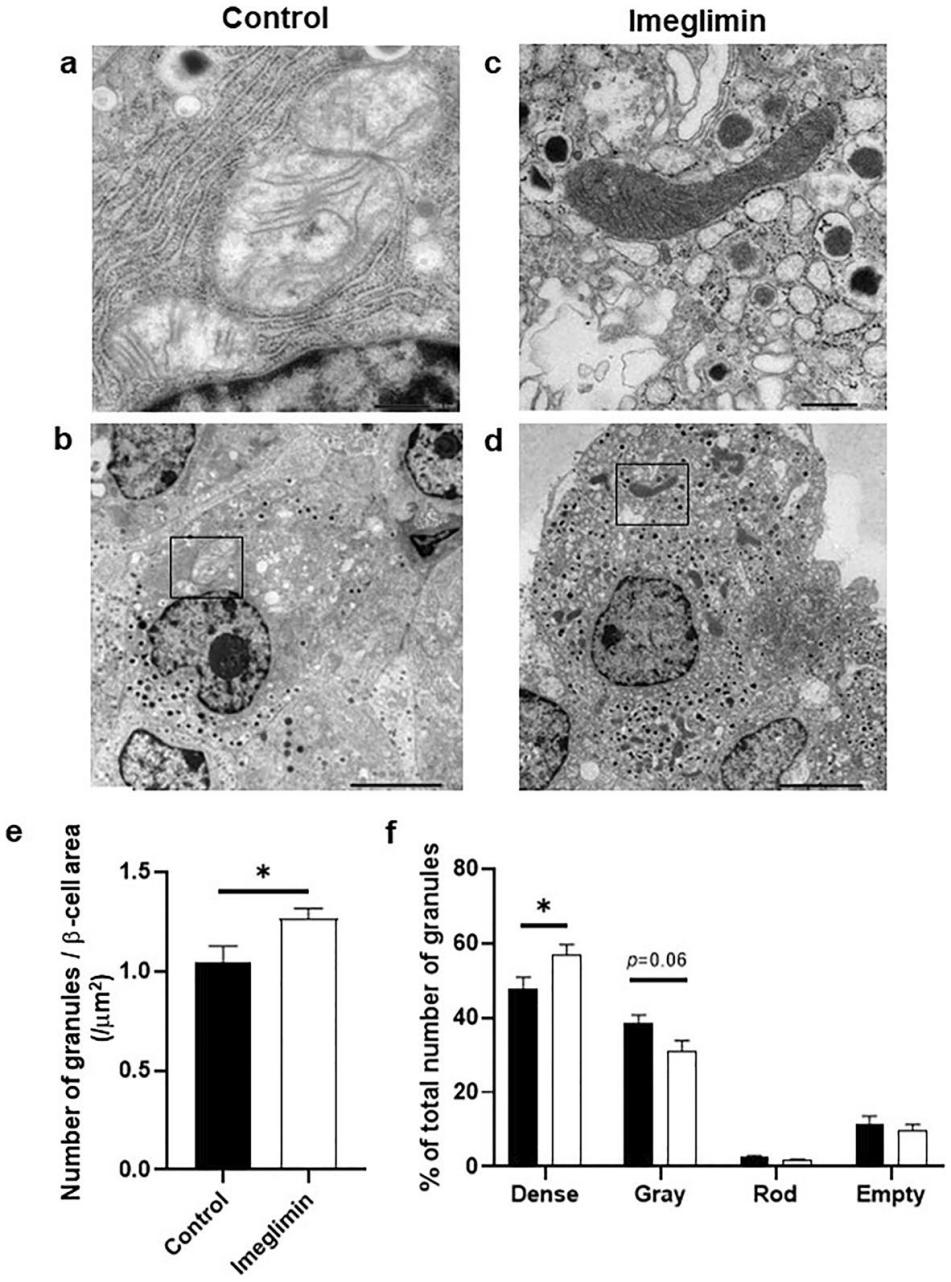

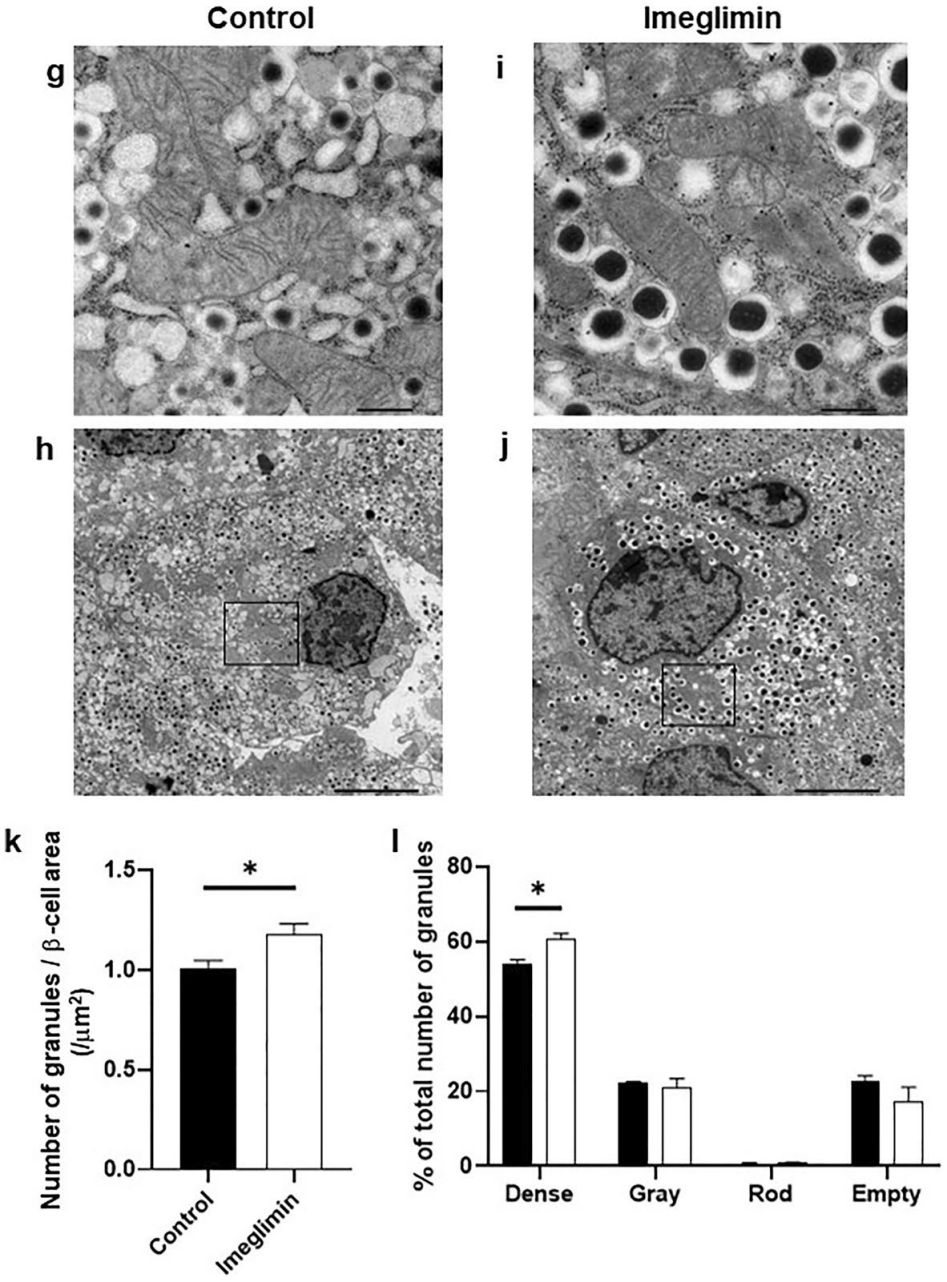
Effects of chronic administration of imeglimin on mitochondrial morphology and the number of insulin granules in β-cells in type 2 diabetic db/db and KK-Ay mice. (**a**–**d**) The mitochondria in β-cells in db/db mice appeared to be swollen and crista structure inside mitochondria was disrupted, which was not observed in db/db mice treated with imeglimin. In addition, the number of insulin granules was preserved in db/db mice treated with imeglimin compared to that without the treatment. (**e**) Quantitative evaluation of the number of insulin granules in β-cells in db/db mice after 4-week treatment with imeglimin. The number of insulin granules in β-cells was significantly higher in the mice treated with imeglimin compared to that without the treatment. (**f**) Quantitative evaluation of insulin secretory granules according to the core; dense, gray, rod-shaped, or empty. (**g**–**j**) The mitochondria in β-cells in KK-Ay mice also appeared to be swollen and crista structure was disrupted, which was not observed in KK-Ay mice treated with imeglimin. In addition, the number of insulin granules was preserved in KK-Ay mice treated with imeglimin. (**k**) Quantitative evaluation about the number of insulin granules in β-cells in KK-Ay mice after 4-week treatment with imeglimin. (**l**) Quantitative evaluation of insulin secretory granules according to the core; dense, gray, rod-shaped, or empty (n = 4–8). Values are the mean ± SEM of data obtained from each group. *p < 0.05. Bar: 0.5 µm (**a**, **c**, **g**, **i**), 5.0 µm (**b**, **d**, **h**, **j**).

First, to examine the possible influence of imeglimin on pancreatic islet mass, we measured islet mass after 4-week treatment with imeglimin in type 2 diabetic db/db and KK-Ay mice. As shown in Supplemental Fig. 3, however, there was no difference between control and imeglimin group in db/db and KK-Ay mice. Next, to evaluate the possible influence of imeglimin on mitochondrial morphology and the number of insulin granules in β-cells, we observed β-cells in more detail using an electron microscope. Since it was previously reported that imeglimin improved mitochondria function, we evaluated the possible effects of imeglimin on morphology in β-cell mitochondria. As shown in Fig. 3a–d, there was marked difference in morphology in β-cell mitochondria in obese type 2 diabetic db/db mice treated with imeglimin for 4 weeks compared to untreated mice. The mitochondria in β-cells in untreated db/db mice appeared to be swollen and crista structure inside mitochondria was disrupted, which was not observed in db/db mice treated with imeglimin. In addition, the number of insulin granules was lower in β-cells of db/db mice compared to that in non-diabetic db/m mice (Supplemental Fig. 4). As shown in Fig. 3a–d, however, the number of insulin granules was preserved in db/db mice treated with imeglimin compared to untreated mice. Next, we performed quantitative evaluation about the number of insulin granules in β-cells and compared the number between with and without 4-week treatment with imeglimin. As shown in Fig. 3e, the number of insulin granules in β-cells was significantly higher in the mice treated with imeglimin compared to that without treatment. Furthermore, we evaluated the possible influence of imeglimin on the characteristics of insulin granules in more detail. As shown in Fig. 3f, the percentage of dense insulin granules was higher after the treatment with imeglimin compared to that without it. In contrast, the percentage of gray insulin granules tended to be lower after imeglimin treatment. There was no difference in percentage of rod-shaped or empty insulin granules between with and without imeglimin treatment. Similar results were obtained in type 2 diabetic KK-Ay mice. As shown in Fig. 3g–j, there was marked difference in morphology in β-cell mitochondria in KK-Ay mice between with and without 4-week treatment with imeglimin. The mitochondria in β-cells in KK-Ay mice also appeared to be swollen and crista structure was disrupted, which was not observed in KK-Ay mice treated with imeglimin. In addition, the number of insulin granules was lower in β-cells of KK-Ay mice compared to that in non-diabetic db/m mice. As shown in Fig. 3g–j, however, the number of insulin granules was preserved in KK-Ay mice treated with imeglimin compared to that without treatment. Furthermore, as shown in Fig. 3k, in quantitative evaluation, the number of insulin granules in β-cells was significantly higher in KK-Ay mice treated with imeglimin compared to that without treatment. Furthermore, we evaluated the possible influence of imeglimin on the characteristics of insulin granules in more detail. As shown in Fig. 3l, the percentage of dense insulin granules was higher after the treatment with imeglimin compared to that without it in KK-Ay mice as observed in db/db mice. There was no difference in the percentage of gray, rod-shaped or empty insulin granules between with and without imeglimin treatment.

### Favorable effects of chronic treatment with imeglimin on apoptotic β-cell death

**Figure 4:**
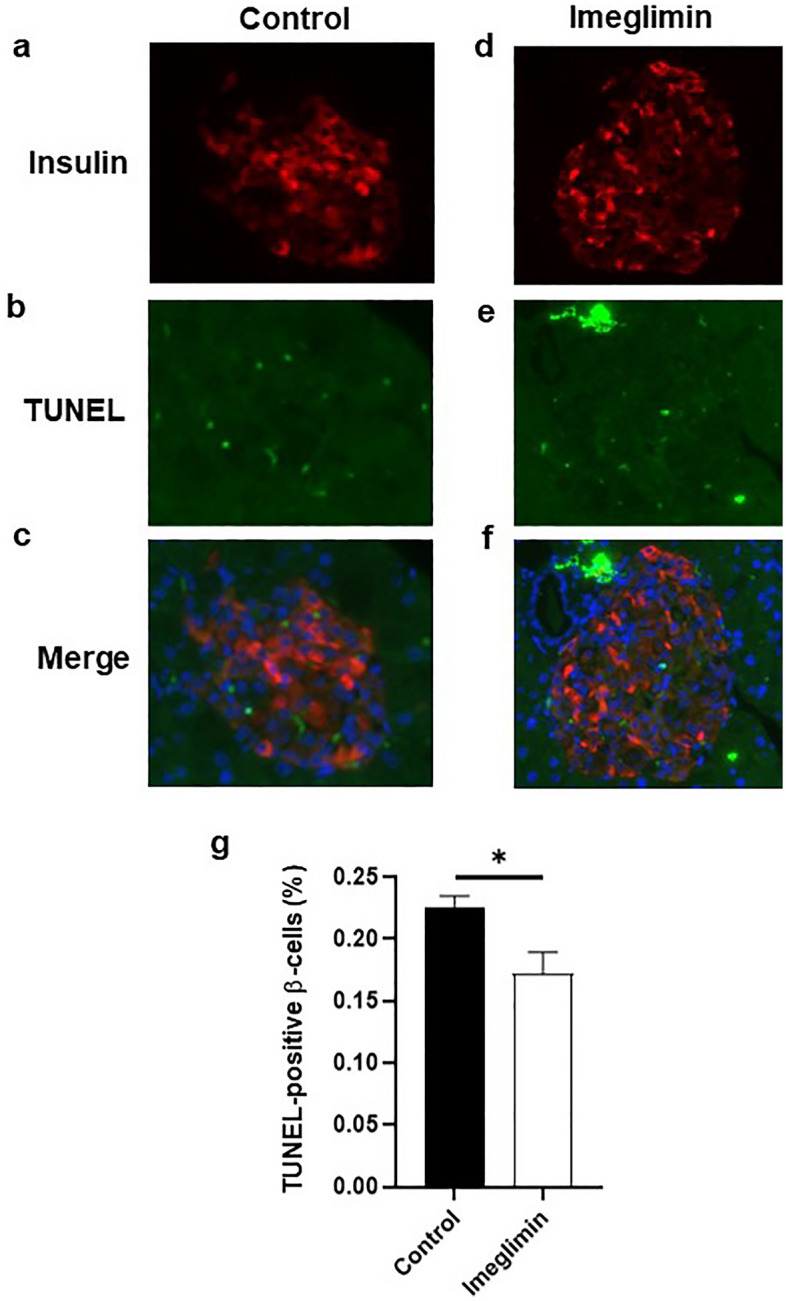

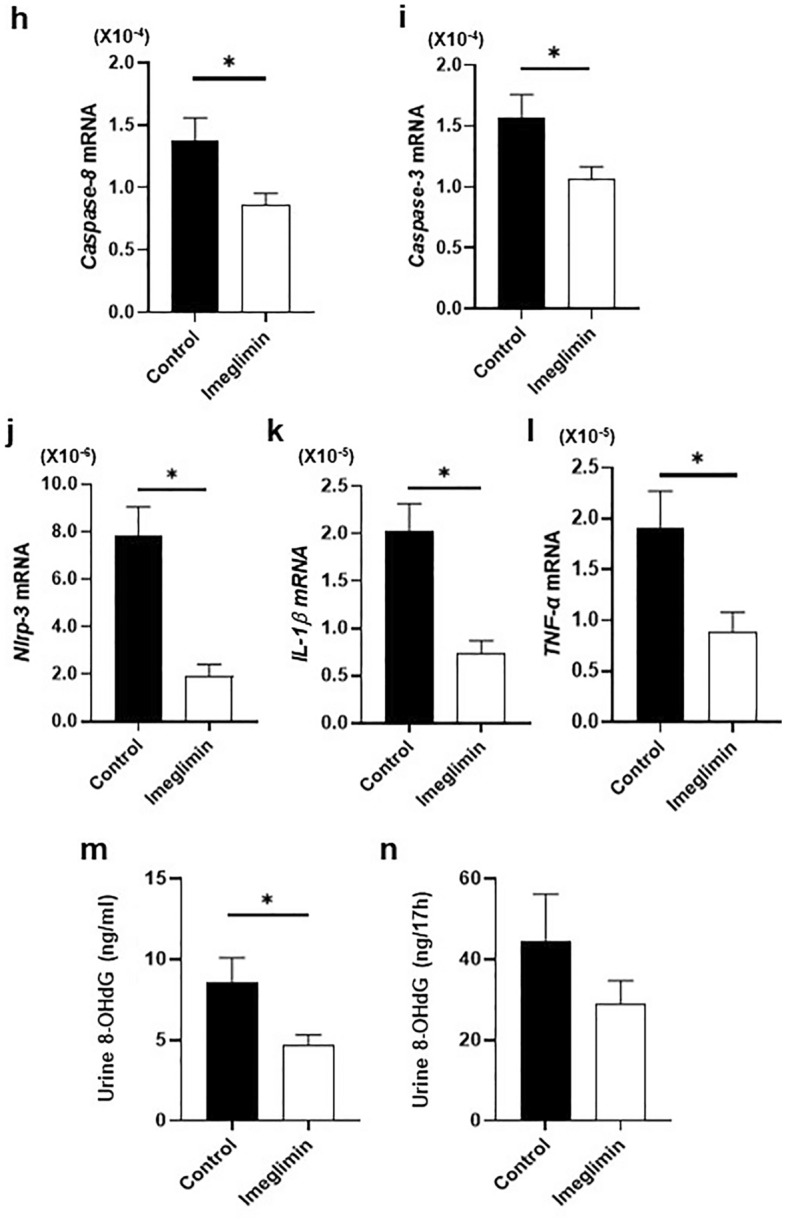
Effects of chronic administration of imeglimin on apoptosis in pancreatic β-cells in obese type 2 diabetic db/db mice. (**a**–**f**) In immunostaining with insulin and TUNEL, TUNEL-positive β-cells were more often observed in the mice without treatment with imeglimin compared to those with the treatment. (**g**) Quantitative evaluation about the effects of imeglimin on the percentage of TUNEL-positive β-cells (n = 5–6). (**h**–**l**) Expression levels of apoptosis- and inflammation-related genes in db/db mice after 4-week treatment with imeglimin. Expression levels of caspases, inflammatory cytokines and NLRP-3 were all significantly lower in the mice treated with imeglimin (n = 9). (**m**–**n**) Effects of imeglimin on oxidative stress in db/db mice. Level of urinary 8-OHdG was lower in diabetic db/db mice treated with imeglumin compared to those without the treatment (n = 4–5). Values are the mean ± SEM of data obtained from each group. *p < 0.05.

Since it is well known that deterioration of β-cell function is closely associated with apoptotic β-cell death, we examined the effects of imeglimin on the percentage of β-cell apoptosis. First, to evaluate the effects of imeglimin on the ratio of apoptosis in β-cells, we performed TUNEL assay in type 2 diabetic db/db mice with and without 4-week treatment with imeglimin. As shown in Fig. 4a–f, in immunostaining with insulin and TUNEL, TUNEL-positive β-cells were more often observed in the mice without treatment with imeglimin compared to those with the treatment. Second, we performed quantitative evaluation about the percentage of TUNEL-positive β-cells and compared the percentage between with and without 4-week treatment with imeglimin. As shown in Fig. 4g, the percentage of TUNEL-positive β-cells was significantly lower in the mice treated with imeglimin compared to that without treatment.

Next, we evaluated expression levels of apoptosis-related genes in db/db mice after 4-week treatment with imeglimin. For example, it is well known that several caspases are closely associated with the process of apoptotic cell death. As shown in Fig. 4h,i, expression levels of caspase3 and caspase8 were all significantly lower in the mice treated with imeglimin compared to those without treatment. It is also known that IL-1β, TNF-α, and NLRP-3 are involved in progression of inflammation and activation of inflammasome. As shown in Fig. 4j–l, expression levels of IL-1β, TNF-α, and NLRP-3 were all significantly lower in the mice treated with imeglimin compared to those without treatment. It is also known that oxidative stress brings about apoptotic cell death. Therefore, we finally evaluated markers of oxidative stress which can induce apoptotic β-cell death. As shown in Fig. 4m–n, level of urinary 8-OHdG was lower in diabetic db/db mice treated with imeglimin compared to those without treatment. Taken together, it is likely that imeglimin reduces apoptotic β-cell death, at least in part, by suppressing inflammation and/or oxidative stress.

## Discussion

In this study, we examined the effect of imeglimin, a new anti-diabetic drug, on pancreatic β-cells using type 2 diabetic mice. First, single-dose administration of imeglimin directly enhanced insulin secretion from β-cells in several models such as type 2 diabetic db/db mice and islets isolated form non-diabetic db/m mice. In addition, imeglimin augmented insulin secretion under a high glucose concentration, but not in a low glucose concentration (Fig. 1c), suggesting that imeglimin enhances insulin secretion in a glucose concentration-dependent manner. Second, chronic treatment with imeglimin enhanced insulin secretory capacity and ameliorated glycemic control in type 2 diabetic db/db mice. Third, evaluation with electron microscopy clearly demonstrated for the first time that imeglimin showed favorable effects on morphology in β-cell mitochondria and substantially increased the number of insulin granules in β-cells. Fourth, imeglimin significantly reduced apoptotic β-cell death together with suppression of apoptosis- and/or inflammation-related gene expression levels and reduction of oxidative stress.

Based on the findings in this study, we think that imeglimin has the following functions in β-cells, namely enhancement of insulin secretion, preservation of the number of insulin granules, recovery of morphology in mitochondria, and reduction of apoptosis. One function of imeglimin is to directly enhance insulin secretion from β-cells. We assume that such enhancement of insulin secretion is presumably due to an increase in intracellular calcium level by imeglimin^17^. Another function is to increase the number of insulin granules in β-cells. It seems that there is some association between these two functions, because it is likely that when the number of insulin granules is preserved in β-cells, imeglimin can more easily facilitate insulin secretion. Another function is to recover the morphological change in β-cell mitochondria. We think that such phenomena are also involved in the above-mentioned functions in β-cells, because recovery of morphological change in β-cell mitochondria likely facilitates ATP production and thereby enhances insulin biosynthesis and secretion. Another function is to reduce apoptotic β-cell death. We think that reduced β-cell apoptosis is presumably due to decreased expression levels of various apoptosis- and/or inflammation-related factors such as several kinds of caspases, inflammatory cytokines, and NLRP-3. Also, we think that decrease of oxidative stress by imeglimin, at least in part, leads to reduction of β-cell apoptotic cell death. In addition, it seems that there is also some association between the reduction of apoptotic β-cell death by imeglimin and the above-mentioned deterioration of β-cell function. It is likely that when apoptotic β-cell death is induced, it is difficult for β-cells to preserve insulin biosynthesis and secretion. Further study would be necessary to unveil the precise association among the above-mentioned effects of imeglimin on β-cells, namely enhancement of insulin secretion, preservation of the number of insulin granules, recovery of morphology in mitochondria, and reduction of apoptosis.

In addition, it is thought that imeglimin shows beneficial effects on insulin target organs such as the liver. Indeed, it was reported that imeglimin improved insulin sensitivity and suppressed glycogenesis presumably due to reduction of triglyceride content, reduction of oxidative stress, and enhancement of mitochondria function in the liver^20^. Therefore, imeglimin exerts protective effects against insulin resistance as well as insufficient insulin secretion both of which are main characteristics of type 2 diabetes mellitus. Such dual effects of imeglimin are promising from the point of glucose toxicity. Under diabetic conditions, when β-cells are chronically exposed to hyperglycemia, β-cell function gradually deteriorates, which progressively reduces insulin biosynthesis and secretion. Such phenomena are known as glucose toxicity. In addition, glucose toxicity is involved in the development of insulin resistance by suppressing insulin signaling in insulin target organs. We assume that imeglimin helps to get out vicious cycle of glucose toxicity. When imeglimin enhances insulin secretion, blood glucose levels are decreased, and glycemic control is improved. Such improvement of glycemic control reduces glucose toxicity and thus mitigates insulin resistance by improving insulin signaling in insulin target organs. Conversely, when imglimin improves insulin sensitivity in insulin target organs, excess insulin secretion from β-cells would be reduced. Under such conditions, β-cell overwork is reduced, and thus β-cell function can be recovered. Therefore, we think that the dual function of imeglimin is promising from the point of glucose toxicity.

There is a limitation in this study. First, the precise mechanism by which imeglimin enhanced glucose-stimulated insulin secretion in this study remains unclear. It has been recently reported, however, that imeglimin increases intracellular calcium concentration through the influx of calcium from the endoplasmic reticulum as well as from the outside of β-cells^17^. Therefore, we assume that the increase in intracellular calcium concentration is involved in the facilitation of insulin secretion by imeglimin. Second, the mechanism by which imeglimin improved β-cell mitochondrial function in this study also remains unclear. It has been shown, however, that imeglimin inactivates respiratory chain complex I and conversely activates complex III in mitochondria, which can lead to mitigate the excess production of reactive oxygen species^20^. Therefore, we assume that the alteration of respiratory chain complex situations is involved in the improvement of mitochondrial function by imeglimin. Finally, while metformin is very frequently used in clinical practice as one of insulin sensitizers, imeglimin is a compound which is modified from metformin and thus is expected to exert favorable effect on insulin sensitivity. Indeed, as described above, imeglimin suppresses glycogenesis in the liver and improves insulin sensitivity^20^. Therefore, it seems that the improvement of insulin sensitivity by imeglimin also contributes to the protection of β-cell function by reducing the excess biosynthesis and/or secretion of insulin from β-cells and thereby mitigating β-cell overwork and debilitation which is often observed under chronic hyperglycemic conditions.

In addition, in this study we used relatively young diabetic mice, which are thought to show rather hyperplastic stage of islets. It is known, however, that this diabetic model shows substantial debilitation of β-cell function and reduction of β-cell mass with increasing age. Considering that most diabetic patients are relatively elder in clinical practice, we think that it would be very important to examine whether imeglimin can show similar favorable effects on β-cells even at an advance stage of diabetes. Indeed, it was reported that several anti-diabetic drugs such as incretin-related drugs and sodium-glucose cotransporter 2 inhibitors showed favorable effects on β-cells at an early stage of diabetes but that such phenomena were not observed at an advanced stage^24–26^. Therefore, it is possible that the effects of imeglimin would be weakened at an advanced stage. In consideration of all things, it would be safe to conclude at this moment that imeglimin shows substantial favorable effects on β-cells at least when it is used at an early stage of diabetes.

In conclusion, imeglimin directly enhances insulin secretion from β-cells, increases the number of insulin granules, exerts beneficial effects on morphology in β-cell mitochondria, and reduces β-cell apoptosis in type 2 diabetic mice, which finally leads to enhancement of glucose-stimulated insulin secretion and amelioration of glycemic control. While the number of subjects with type 2 diabetes mellitus has been increasing worldwide, it has become possible very recently that we can prescribe imeglimin in clinical practice. Clinical trials with imeglimin in type 2 diabetic patients have also recently shown its efficacy and safety of long-term therapy and its effectiveness of combination with other oral anti-diabetic drugs or insulin therapy. Therefore, we hope that the findings in this report would provide useful information to many clinicians as well as researchers that imeglimin would be promising in diabetes care especially for the enhancement of pancreatic β-cell function.

## Materials and methods

### Animals

We used 7-week-old male KK-Ay/TaJcl (KK-Ay) mice, BKS. Cg-m +/+ Lepr db/Jcl (db/m) mice and BKS.Cg- + Lepr db/ + Lepr db/Jcl (db/db) mice that were purchased from Clea, Tokyo, Japan. Animals were housed under controlled ambient conditions and 12 h light and dark cycle. They were given free access to water and standard diet (MF; Oriental Yeast Co., Ltd.) and maintained at 25 °C. They were treated with imeglimin (200 mg/kg, twice daily oral gavage) or 0.5% carboxymethyl cellulose (CMC) for 4 weeks. Imeglimin was kindly provided by Sumitomo Pharma. The drug dose was set based on a previous report^20^. Body weights, food intake and water intake were measured every week until the end of experiment. The study was approved by the animal use committee of Kawasaki Medical School (NO.19-103) and conducted in compliance with the animal use guidelines of Kawasaki Medical School.

### Measurement of biochemical markers

Blood samples were collected from tail vein, and blood glucose levels were measured using a glucometer (Glutest Mint; Sanwa Kagaku Kenkyusho Co, Ltd, Japan) every week. Serum total cholesterol and triglyceride were measured enzymatically using the Wako LabAssay (Wako Pure Chemical Industries, Japan). Urine was collected using metabolic cage at 11 weeks age, and levels of 8-OHdG were measured using ELISA kit (Japan Institute for the Control of Aging, NIKKEN SEIL Co, Ltd, Japan).

### Oral glucose tolerance test (OGTT)

After 16 h fasting, animals were given a single dose of imeglimin, and d-(+)-glucose (1 g/kg BW) was administered orally for OGTT at 7 weeks old. We performed OGTT again after 4-week treatment with imeglimin. Blood samples were collected at 0, 15, 30, 60, 90 and 120 min and blood glucose levels were measured using Glutest Mint. Serum insulin levels were determined using a mouse insulin ELISA kit (Morinaga, Tokyo, Japan). The experiment was conducted in accordance with the previous report^26^.

### Insulin tolerance test (ITT)

Insulin tolerance test was performed by intraperitoneal injection (db/db mice 2 U/kg BW, KK-Ay mice 1 U/kg BW) of human regular insulin (Novo Nordisk, Bagsvaerd, Denmark) after 5-h fasting. Blood glucose levels were monitored at 0, 15, 30, 60, 90, 120 min after insulin injection.

### Pancreatic islet isolation

Isolation of islets from the pancreas of the mice was conducted as previously described^27^. In brief, islets were isolated after clamping the common bile duct at its entrance to the duodenum. 2.5 ml of Hanks’ Balanced Salt Solution (HBSS) (Sigma, St Louis, MO, USA) containing 0.6 mg Liberase TL (Roche Diagnostics, Tokyo, Japan) and 25 mmol/l HEPES was injected into the duct. The swollen pancreas was removed and incubated at 37 °C for 24 min. The pancreatic tissue was then dispersed by pipetting and washed twice with ice-cold HBSS containing 25 mmol/l HEPES and 10% (wt/vol.) FBS. Thereafter, the islets were manually picked up under a stereoscopic microscope and used immediately for the experiments.

### Glucose stimulated insulin secretion (GSIS)

Size matched pancreatic islets isolated from mice were prepared (five pancreatic islets/tube) and pre-incubated in HEPES buffer. The supernatant fraction was replaced with glucose solution (either 3 mmol/l or 16.7 mmol/l) and the mixture was incubated for additional 60 min at 37 °C as previously reported^26,27^. The supernatant fraction and islets were stored at – 80 °C until use.

### Immunofluorescence

According to the previously established method^10,11^, isolated pancreases were fixed overnight with formaldehyde at 4 °C. Tissues were routinely processed for paraffin embedding and 4 μm sections were cut and mounted on silanized slides. To investigate cell apoptosis in pancreatic islets, TUNEL assay was performed using a DeadEnd Fluorometric TUNEL System (DeadEnd, Promega, Madison, WI, USA).

### Electron microscopy

Isolated islets were fixed for 2 h in 2.5% (vol./vol.) glutaraldehyde buffered to pH 7.4 with phosphate buffer and treated with osmium tetroxide for 1 h at 4 °C. The tissues were dehydrated with graded concentration ethanol and then embedded in Epon 812 (Nakarai, Kyoto, Japan). Thin sections were cut with a Leica Ultracut S (Leica Microsystems, Tokyo, Japan) with a diamond knife and stained with uranyl acetate followed by lead citrate^28^. Electron micrographs were taken with a JEOL JEM-1400 electron microscope (JEOL, Tokyo, Japan) operated at 80 kV. Dense core, light core (gray), atypical (rod-shaped) and empty granules were manually counted and quantified^29^. Number of granule/β-cell area (/μm^2^) = (dense granules + gray granules)/β-cell area.

### Morphometric analysis

The image analysis software NIH Image (version 1.61; http://rsbweb.nih.gov/ij/) was used to calculate the pancreas area and islet area. Using a total of nine sections (three sections from three different areas of the pancreas) for each group of mice, islet mass was estimated via the following formula: mass (mg) = average of islet area per section / average of pancreas area per section × weight of pancreas.

### RNA isolation and real time PCR

Pancreatic islets isolated from mice treated with imeglimin were incubated with 1 mM imeglimin for 24 h. Total RNA extraction was performed using an RNeasy Mini kit (QIAGEN, Valencia, CA) according to the manufacturer’s instructions. cDNA was produced from mRNA using TaqMan reverse transcription reagents (Applied Biosystems, Foster City, CA). Quantitative PT-PCR was conducted using a StepOne Real-Time PCR system (Applied Biosystems). To quantify gene expression, the 2^−ΔCT^ was calculated using 18-sr as an internal control. Primer sequences used for real time PCR are presented in Supplementary Table 1.

### Statistical analysis

All data were analyzed and expressed as the mean ± standard error of the mean. Differences between two groups were tested for statistical significance using Student’s t-test. p values less than 0.05 were considered to indicate a statistically significant difference.

### Declaration of study performance

The study is reported in accordance with ARRIVE guidelines.

## Supplementary Information

Supplementary Legends.

Supplementary Figure 1.

Supplementary Figure 2.

Supplementary Figure 3.

Supplementary Figure 4.

Supplementary Table 1.

## Data Availability

The datasets generated and/or analyzed during the current study are available from the corresponding author upon reasonable request.
