# Supplementary material for: Imeglimin exerts favorable effects on pancreatic β-cells by improving morphology in mitochondria and increasing the number of insulin granules
Source: Sci Rep. 2022 Aug 2;12:13220. doi: 10.1038/s41598-022-17657-3 (PMC9345869; doi:10.1038/s41598-022-17657-3)
Supplement: Supplementary file 1 — Supplementary Legends. [file 41598_2022_17657_MOESM1_ESM.docx]

**Supplemental Figure Legends**

Supplementary Fig. 1. Effect of chronic administration of imeglimin on insulin resistance. There was no difference in blood glucose levels between the two groups after insulin tolerance test. (a) db/db mice, (b) KK-Ay mice. Control: black circle, imeglimin: white circle, n=6-7. Values are the mean ±SEM of data obtained from each group.

Supplementary Fig. 2. Body weight, serum total cholesterol and triglyceride levels before (7-week-old) and after the treatment (11-week-old). (a) Body weight, (b) total cholesterol, (c) triglyceride in db/m mice. (d) Body weight, (e) total cholesterol, (f) triglyceride in db/db mice. (g) Body weight, (h) total cholesterol, (i) triglyceride in KK-Ay mice. Control: black bar, imeglimin: white bar, n=6-10. Values are the mean ±SEM of data obtained from each group.

Supplementary Fig. 3. Pancreatic islet mass after 4-week treatment in obese type 2 diabetic db/db and KK-Ay mice. There was no difference between control and imeglimin group in db/db mice (a) and KK-Ay mice (b). n=4-6. Values are the mean ±SEM of data obtained from each group.

Supplementary Fig. 4. Electron microscopy in β-cells in non-diabetic db/m mice. Black arrows: mitochondria, white arrows: dense granule, black arrowheads: gray granule, white arrowheads: rod-shaped granule. Bar: 0.5 μm (a), 5.0 μm (b).
