# Supplementary figures and images for: Imeglimin exerts favorable effects on pancreatic β-cells by improving morphology in mitochondria and increasing the number of insulin granules
Source: Sci Rep. 2022 Aug 2;12:13220. doi: 10.1038/s41598-022-17657-3 (PMC9345869; doi:10.1038/s41598-022-17657-3)

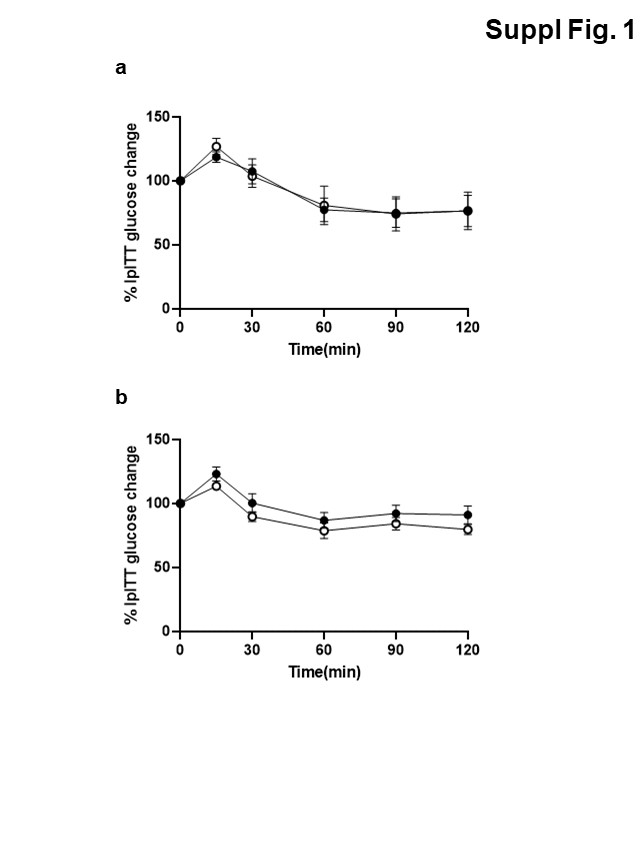

Supplement: Supplementary file 2 — Supplementary Figure 1. [file 41598_2022_17657_MOESM2_ESM.jpg]

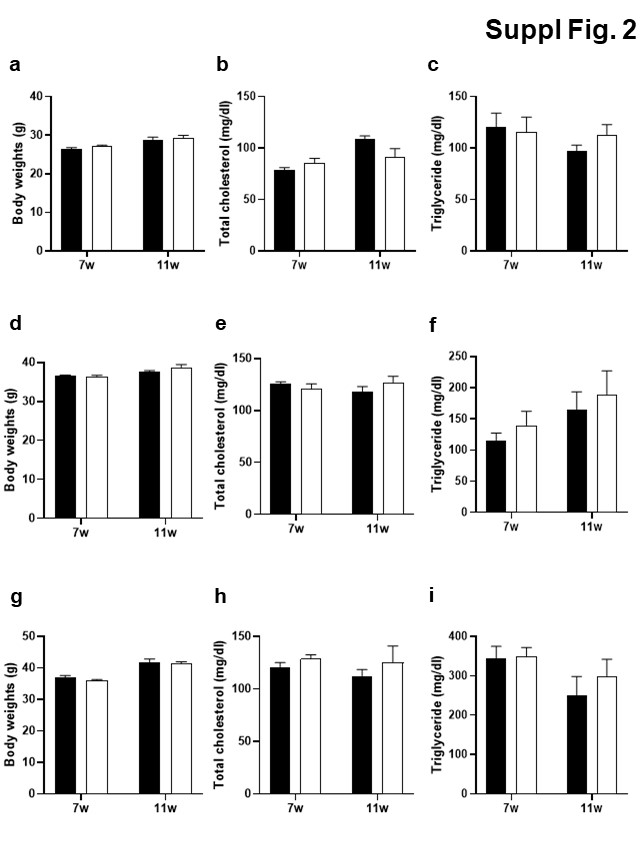

Supplement: Supplementary file 3 — Supplementary Figure 2. [file 41598_2022_17657_MOESM3_ESM.jpg]

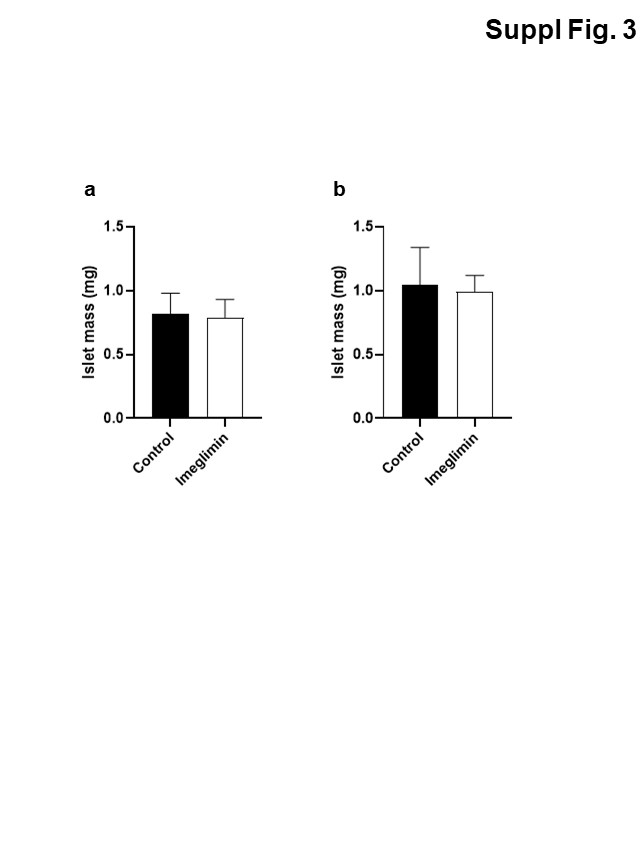

Supplement: Supplementary file 4 — Supplementary Figure 3. [file 41598_2022_17657_MOESM4_ESM.jpg]

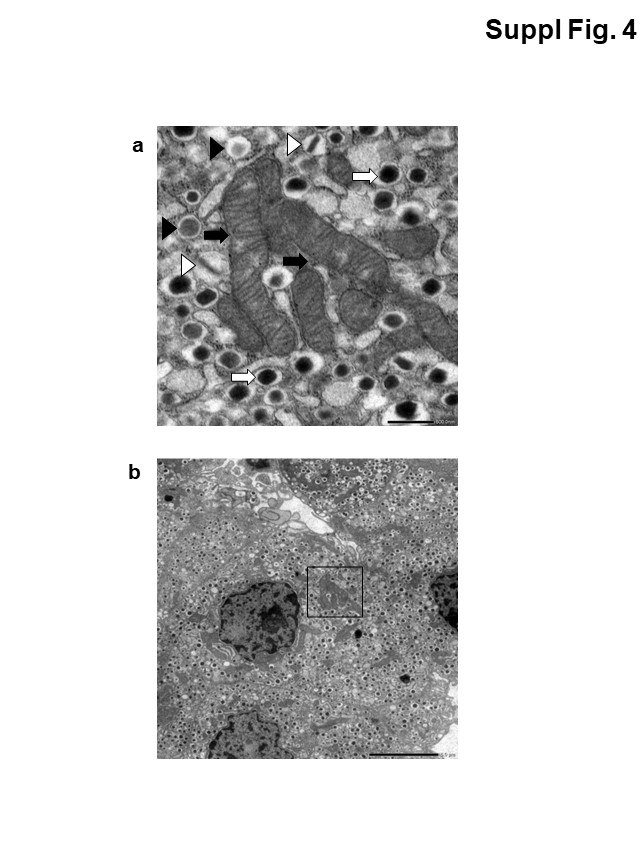

Supplement: Supplementary file 5 — Supplementary Figure 4. [file 41598_2022_17657_MOESM5_ESM.jpg]
