# Supplementary material for: Imeglimin exerts favorable effects on pancreatic β-cells by improving morphology in mitochondria and increasing the number of insulin granules
Source: Sci Rep. 2022 Aug 2;12:13220. doi: 10.1038/s41598-022-17657-3 (PMC9345869; doi:10.1038/s41598-022-17657-3)
Supplement: Supplementary file 6 — Supplementary Table 1. [file 41598_2022_17657_MOESM6_ESM.docx]

Supplementary Table 1：Primer sequences of forward and reverse primers for real-time PCR

| Genes | Forward | Reverse |
| --- | --- | --- |
| 18sr | GCGCTTCCTTACCTGGTTGAT | GCCATTCGCAGTTTCACTGTAC |
| Caspase-8 | GAGATCCTGTGAATGGAACCTGGTA | GTTCACGCCAGTCAGGATGCTA |
| Caspase-3 | CTGGACTGTGGCATTGAGACA | CAGCCTCCACCGGTATCTTC |
| NLRP-3 | CCTTGGACCAGGTTCAGTGT | AGGCAGCAGTTCACCAGTCT |
| IL-1β | TGGTGTGTGACGTTCCCATTA | CGACAGCACGAGGCTTTTTT |
| TNF-α | TGATCCGCGACGTGGAA | ACCGCCTGGAGTTCTGGAA |
